# Natural Cleanliness of Plants and Animals

**Published:** 1904-03

**Authors:** 


### NATURAL CLEANLINESS OF PLANTS AND ANIMALS.

WHEN the pious Heber wrote, "Every prospect pleases, only man is vile," who knows but that his reflective gaze wandered from the frowsy habilaments of a passing knight of the road, blotting the rich smoothness of an English landscape, back to his study where it encountered the glittering corselet of the cockroach, as she emerged immaculate from a dusty cranny? In whatever way the contrast arose in his mind, it is evident to every one that plants and animals, which go to make up a large part of that which we call nature, are eminently cleanly and orderly and neat.

The green of the struggling grass that thrusts itself upward through its environment of filth is as bright and lustrous as though polished by hand. No dust clings to it that a chance shower or a vagrant breeze does not blow away. The leaves of the trees seem to shake themselves as a woman shakes her skirts, as though shuddering with disgust at chance contamination.

Despite unseemly habits and strange dietetic preferences, what can be more scrupulous of its personal cleanliness than the common blow-fly? It is amusing to watch with what punctilious care each wing is bent and brushed, the eyes and head rubbed free from dust, and nothing omitted to make the *toilette* complete and satisfactory before further questing for food.

Is there a finer example in all nature of rising superior to surroundings than that offered by the Egyptian emblem of immortality, the obscene scavenger beetle, doomed like Sisyphus forever to roll a ball up hill. Never did Sir Galahad more assiduously burnish his suit of mail than does the tumblebug his wing-cases and horny integuments. He arises from his unsavory bed and board as free from change as Shadrach, Meshach and Abednego, but, unlike those intrepid believers, with a very decided smell upon his garments.

The yellow butterflies that flutter and cluster about a mud-puddle in a country road are as spick and span and unruffled as though they were dressed up for the occasion and were waiting to make their debut in the glass case of a lepidopterous collection. When the detail is brought out by a hand-lens, their feathers are as regular and symmetrical as the curled plumes of an ostrich in a milliner's window.

While the good Heber probably referred to the decadence of man's moral nature, his expression is appropriate to man's physical state, when it is held in comparison with that of animals. Man possesses none of that self-cleansing capacity that marks the animal. Ceruminous, sudoriparous, carious, man begins to decay ere yet the mother's milk has dried upon his chin, and without man-given soap and God-given water he would soon generate an effluvium beside which the pungent emanations of the ball-bearing beetle would be as a spice-laden breeze from Araby the Blest.

To hide and disguise this vileness he must borrow the natural perfumes of animals and plants, and often in such excess that, oiled and curled like an Assyrian bull, his essences make the live air sick.

To get the benefit of the contrast, compare the wholesome sleekness of a young heifer with some musty shellback, from whose legs the dirt could be chipped off with a scaling-hammer. In such a beauty contest between man and brute, the heir of the ages gets second money. In the matter of food, man will rush in where animals fear to tread. A dog slobbers freely at the taste of a tick or a fly, and yet a returned missionary from Africa at one of his lectures passed around a loaf of native bread made of dried flies, and noticed, when it had been returned him, that half of it was nibbled away. The Schnepfen dröch of the North Prussians is not good to muse over, while the various kinds of putrid cheese eaten with so much avidity are well suited to the peculiar taste of the hyena woman Amina, whose necrophilous cravings were satisfied by night, while by day she delicately ate rice with a bodkin. In the matter of cheese, one is reminded of Dean Swift's Tom Mullinix and Dick:

> "Tom, eating rotten cheese, did say,
> 'Like Sampson, I my thousands slay.'
> 'Yes,' answered Dick, 'That is quite true,
> And with the selfsame weapon, too.'"

But it is useless to attempt a narration of the abominations which man will eat and a dog will wallow upon. The point of these vagrant remarks, if a point be discoverable, is that animals and plants are intrinsically cleaner than man, and "unless above himself he can uplift himself, how poor a thing is man."

